# RPE Cells Engulf Microvesicles Secreted by Degenerating Rod Photoreceptors

**DOI:** 10.1523/ENEURO.0507-19.2020

**Published:** 2020-05-21

**Authors:** Philip Ropelewski, Yoshikazu Imanishi

**Affiliations:** Department of Pharmacology, Case Western Reserve University School of Medicine, Cleveland, Ohio 44106

**Keywords:** blindness, extracellular vesicles, phagocytosis, retinitis pigmentosa, rhodopsin, RPE cells

## Abstract

Rhodopsin is mislocalized to the inner segment plasma membrane (IS PM) in various blinding disorders including autosomal-dominant retinitis pigmentosa caused by class I rhodopsin mutations. In these disorders, rhodopsin-laden microvesicles are secreted into the extracellular milieu by afflicted photoreceptor cells. Using a *Xenopus laevis* model expressing class I mutant rhodopsin or Na^+^/K^+^-ATPase (NKA) fused to Dendra2, we fluorescently labeled the microvesicles and found retinal pigment epithelial (RPE) cells are capable of engulfing microvesicles containing rhodopsin. A unique sorting mechanism allows class I mutant rhodopsin, but not NKA, to be packaged into the microvesicles. Under normal physiological conditions, NKA is not shed as microvesicles to the extracellular space, but is degraded intracellularly. Those studies provide novel insights into protein homeostasis in the photoreceptor IS PM.

## Visual Abstract

**Figure:**
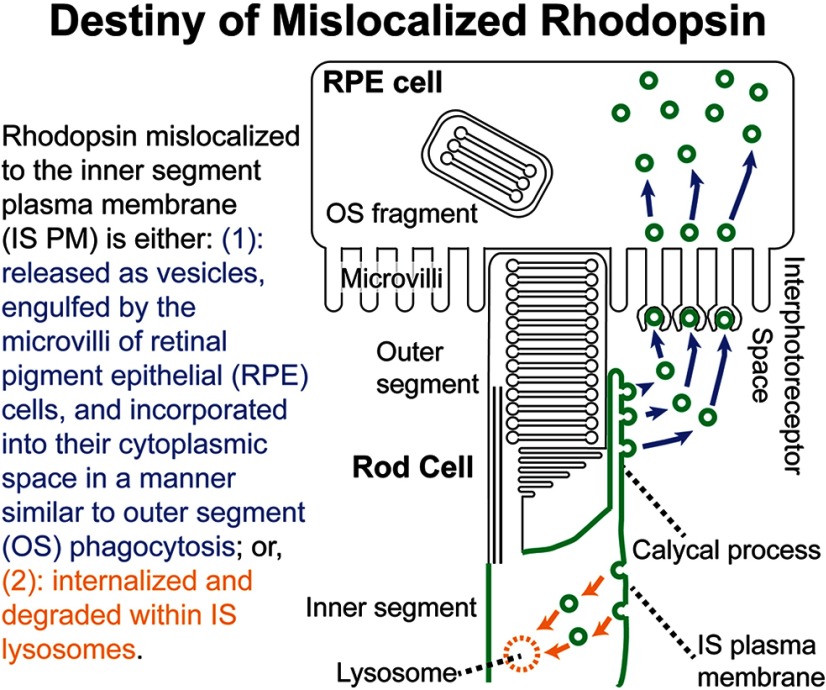

## Significance Statement

Rhodopsin mislocalization is a common cause of photoreceptor degeneration in inherited blinding disorders. In this study, we describe a novel mechanism of removing rhodopsin molecules from inner segment plasma membrane (IS PM). Mislocalized rhodopsin is packaged into microvesicles, which are secreted into the interphotoreceptor space and cleared via engulfment by retinal pigment epithelial (RPE) cells. While IS PM-mislocalized rhodopsin is specifically packaged into microvesicles, Na^+^/K^+^-ATPase α-subunit, an IS PM resident protein, was not sorted into vesicles under either pathologic or normal physiological conditions. Interaction between photoreceptor and RPE cells is critical for maintaining visual function, and its alteration can lead to compromised vision. This study provides novel insights into photoreceptor–RPE cell interaction in inherited blinding disorders.

## Introduction

Photoreceptor and retinal pigment epithelial (RPE) cells evolved a unique symbiotic relationship to maintain the structure and function of the photoreceptive outer segments (OSs). Each day, ∼5–10% of the OS is shed and engulfed by RPE cells, which digest the components—the majority of which is rhodopsin—in phagosomes located within the cytoplasm (Young, 1967; Kevany and Palczewski, 2010). This relationship is disrupted in retinal ciliopathies in which the majority of rhodopsin molecules are no longer destined to the OSs and instead mislocalize to the inner segment (IS) plasma membrane (PM; Sung et al., 1994; Li et al., 1996; Nishimura et al., 2004; Deretic et al., 2005; Adams et al., 2007; Concepcion and Chen, 2010; Hollingsworth and Gross, 2013; Nemet et al., 2015; Imanishi, 2019). In various animal models exhibiting rhodopsin mislocalization, rod photoreceptors expel rhodopsin-laden vesicles, which accumulate in the interphotoreceptor space (Li et al., 1996; Hagstrom et al., 1999; Concepcion and Chen, 2010; Lodowski et al., 2013). The interphotoreceptor space is in constant contact with RPE microvilli, which are optimally positioned for phagocytic activities (Strauss, 2005). Increasing evidence suggests that various neurons shed vesicles as means of communication and to remove unwanted materials under neurodegenerative conditions (Nagarajah, 2016; Fowler, 2019). More recently, RPE cells have been reported to take up extracellular vesicles in an *in vitro* cell culture model (Nicholson et al., 2020). Thus, as the first step in understanding the *in vivo* function of these photoreceptor-derived vesicles, we asked whether RPE cells are capable of engulfing them in a manner analogous to OS phagocytosis. Such studies will shed light on the symbiotic relationship between RPE and photoreceptors under disease states.

Unlike the degradation of OS membrane proteins, which has been relatively well-characterized (Strauss, 2005; Kevany and Palczewski, 2010), little is known about the degradation of IS PM proteins, which lack access to the RPE cells. Thus, we have initiated an effort to understand the renewal of IS PM proteins, especially focusing on class I mutant rhodopsin. In addition to the vesicle-mediated removal described above, mislocalized class I mutant rhodopsin is degraded intracellularly: once reaching the IS PM, mislocalized rhodopsin becomes internalized and subsequently degraded by lysosomes (Ropelewski and Imanishi, 2019). The IS PM component Na^+^/K^+^-ATPase (NKA) plays a critical role in maintaining both the dark current of photoreceptor cells (Yau and Baylor, 1989) and interactions between bipolar and photoreceptor cells (Molday et al., 2007; Friedrich et al., 2011). The lysosome-mediated removal of class I mutant rhodopsin induces co-internalization and co-degradation of native NKA, compromising the structure and function of rod photoreceptors (Ropelewski and Imanishi, 2019). Another IS PM protein, HCN1 channel, plays a role in the normal electrophysiological response of photoreceptor cells, and its deficiency worsens the symptoms of retinitis pigmentosa (Schön et al., 2016). HCN1 contains a di-arginine ER retention signal that negatively regulates PM transport (Pan et al., 2015a), which is suggestive of ER-associated degradation before exiting the ER and reaching the PM. This mechanism appears to be important for regulating the expression level of HCN1 at the level of IS PM (Pan et al., 2015b). Despite improved understanding of HCN1 degradation during biosynthesis or NKA degradation under pathologic states, it is currently unknown whether and how rod photoreceptors coordinate intracellular and intercellular mechanisms for the degradation of endogenous IS PM proteins under normal physiological conditions.

In this article, we investigated the fate of microvesicles shed by rod photoreceptor cells expressing class I mutant rhodopsin. Toward that goal, we used a genetic labeling technique that allowed us to clarify the origin and destination of these secreted vesicles. Then we asked whether there is a sorting mechanism for packaging specific IS PM proteins to the microvesicles. We accomplished this by comparing IS PM-mislocalized rhodopsin and a major IS PM resident protein, NKA. To comprehensively understand the mode of renewing NKA, we compared the extracellular and intracellular mechanisms of degrading NKA. Those studies will provide novel insights into the protein homeostasis of rod photoreceptor cells both in pathologic and normal physiological conditions.

## Materials and Methods

*Animals.* All animal procedures were approved by the Institutional Animal Care and Use Committee at Case Western Reserve University. Adult female and male frogs involved in transgenesis were purchased from Nasco and housed at 16°C under a 12 h light/dark cycle. All tadpoles used for experiments were housed at 16°C in 24 h of darkness. Tadpoles were fed spirulina (https://nuts.com). Both male and female tadpoles were used for all experiments.

*Reagents.* Unless otherwise specified, all reagents were purchased from either Thermo Fisher Scientific or Sigma-Aldrich.

*Molecular cloning.* DNA expression vectors were generated by standard methods combining PCR, DNA recombination, and site-directed mutagenesis. The expression vector containing the *Xenopus* opsin promotor (XOP) followed by GFP-NKAα3 (Laird et al., 2015) was modified. The vector contained an AgeI site upstream of GFP-NKAα. Site-directed mutagenesis was used to create an additional AgeI site after the region encoding GFP; the GFP coding region was then removed using AgeI enzyme and replaced with cDNA encoding Dendra2 fluorescent protein. The resulting DNA fragment was introduced downstream of XOP and upstream of NKAα using In-Fusion Cloning Kit (TaKaRa). The plasmid vectors containing XOP-Rho-Dend2-1D4 and XOP-Rho_Q344ter_-Dend2 were previously generated (Lodowski et al., 2013). All the vectors contained polyadenylation signals following the coding and noncoding regions.

*Transgenesis of* Xenopus laevis. Transgenic *Xenopus laevis* were produced using the intracytoplasmic sperm injection method following the previously published procedure and screened for the presence of Dendra2 fluorescence (Sparrow et al., 2000; Smith et al., 2006; Lodowski et al., 2013). In order to prevent photobleaching, light-dependent photoreceptor degeneration, and unintended photoconversion of fluorescent protein Dendra2, tadpoles were reared in 24 h darkness.

*Immunohistochemistry. X. laevis* retinal sections (12 μm) were prepared and stored at −80°C, as described previously (Lodowski et al., 2013; Ropelewski and Imanishi, 2019). Slides were removed from −80°C storage conditions and warmed using a 37°C incubator (CCC 0.5d, Boekel Scientific). After thawing the sections, all of the following procedures were conducted at room temperature. Individual sections were encircled by an ImmEdge Pen (Vector Laboratories). Sections were then rehydrated and blocked in a 1.5% normal goat serum diluted in PBS solution (137 mm NaCl, 2.7 mm KCl, 8 mm Na_2_HPO_4_, 2 mm KH_2_PO_4_) for 30 min. Blocking solution was then removed, and primary antibody mouse anti-NKAα (1:100; M7-PB-EIX, Santa Cruz Biotechnology) diluted in PBS containing 0.1% Triton X-100 (PBST) was added; sections were incubated for ∼14 h and then washed three times with PBST for 10 min each. After the washes, sections were incubated in PBST containing phalloidin conjugated with Alexa Fluor 633 (1:100; catalog #A22284, Thermo Fisher Scientific), Hoechst 33342 dye (1:500), and goat anti-mouse IgG conjugated either with Alexa Fluor 488 (1:100; catalog #115–545-166, Jackson ImmunoResearch) or with Cy3 (1:100; catalog #115–165-166, Jackson ImmunoResearch) for 1 h followed by three additional 10 min washes with PBST. After these washes, individual sections were mounted with a coverslip and one drop of VECTASHIELD mounting medium (Vector Laboratories). Nail polish (Electron Microscopy Sciences) was used to seal the coverslip, and the sections were dried for >30 min before imaging.

*Preparation of retina explant for confocal imaging.* Tadpoles were killed, decapitated, and their eyes were removed as described previously (Ropelewski and Imanishi, 2019). The RPE layers were separated from the retinas, which were immediately placed into a glass-bottom dish (catalog #P35G-1.5–14-C, MatTek) containing modified Wolf medium (d-glucose, 700 mg/L, 30 mm NaHCO_3_, 55% MEM, 31% sodium-free BBS, 10% FBS; Lodowski et al., 2013). Equilibration of Wolf medium was achieved by incubating the dish containing the medium in an incubation chamber (Tokai Hit) supplied with 5% CO_2_ and 95% O_2_ (Airgas; Nemet et al., 2014). The glass-bottom dishes were coated with Cell-Tak Cell and Tissue Adhesive (Corning). A circular glass coverslip (12 mm diameter; 0.13–0.17 mm thickness) was used to seal and flatten the retinas before imaging.

*Confocal microscopy, image analysis, quantification, and statistical analysis.* All images were acquired using a Leica TCS SP2 laser-scanning confocal/multiphoton microscope system equipped with the following four lasers for excitation: 488 nm argon ion, 543 nm HeNe, 633 nm HeNe, and tunable Chameleon XR Ti:Sapphire laser (Leica Microsystems), as described previously (Ropelewski and Imanishi, 2019). An HCX PL APO CS 40.0 × 1.25 oil UV objective lens was used for imaging. Diameters of vesicular structures were measured using the ImageJ line function tool (Schneider et al., 2012). For irregularly shaped vesicles, which were approximated as ovals, the diameter was calculated by drawing a line at a 45° angle from the cross section to focal points on the edges of the structure. This calculated distance between the focal points was used as the approximated diameter. For the quantitative studies involving drug treatments, the same laser power and imaging condition were used for all the samples. The cytoplasmic space was highlighted manually, and its mean fluorescence intensity measured using the ImageJ freehand selection tool (Schneider et al., 2012). The background noise was determined from blank space where no cells were observed and was subtracted from the measurements. For quantification of cytoplasmic Dend2-NKAα, statistical outliers were identified by the interquartile range method and removed (Kokoska and Zwillinger, 2000). An estimation plot graph was generated via Prism software version 8.4.0 (GraphPad Software). In retina explants from 9 d postfertilization (DPF) animals, we quantified the number of vesicles associated with individual rod cells. For this purpose, vesicles within 2 μm of each rod photoreceptor cell surface were considered to be associated with the cell. Each vesicle was considered to be associated with only one cell. Unless otherwise noted, the quantitative data are represented as the mean ± SD (*n *=* *4 animals or structures thereof). In comparing two populations, *p <* *0.001 by Student’s *t* test was considered statistically significant.

## Results

### IS-mislocalized rhodopsin is secreted and uptaken by RPE cells

**Figure 1.**
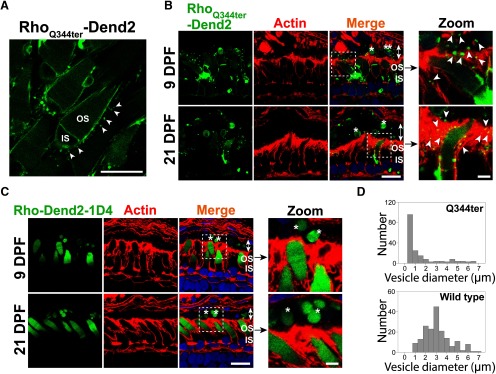
Class I mutant rhodopsin is released as microvesicles before being engulfed by RPE cells. ***A***, Live retina explant was imaged at 9 DPF. Vesicles (arrowheads) were observed in the extracellular space surrounding rods expressing class I mutant rhodopsin, Rho_Q344ter_-Dend2. ***B***, Cross sections of retinas expressing Rho_Q344ter_-Dend2 (Dend2, green) labeled with phalloidin-Alexa Fluor 633 (Actin, red) and Hoechst 33342 dye (Nuclei, blue). Microvesicles containing Rho_Q344ter_-Dend2 were in close contact with the actin filaments of the RPE microvilli (Zoom, arrowheads) and within RPE cytoplasmic space (Merge, indicated by double-headed arrows). Microvesicles were observed both at 9 DPF (top panels) and 21 DPF (bottom panels). Large OS fragments (single asterisk) or cell bodies (double asterisks) also existed within the RPE cell layer (double-headed arrows). ***C***, Cross sections of retinas expressing Rho-Dend2-1D4 (Dend2, green) were labeled with phalloidin-Alexa Fluor 633 (Actin, red) and Hoechst 33342 dye (Nuclei, blue). OS fragments (Zoom, single asterisk) containing Rho-Dend2-1D4 were visible in cytoplasmic space (Merge, double-headed arrows) of the RPE cells. Retinas were imaged either at 9 DPF (top panels) or at 21 DPF (bottom panels). ***D***, Size distribution of green fluorescent structures/vesicles found in the RPE of animals expressing Rho_Q344ter_-Dend2 (Q344ter, based on 180 structures from *n *=* *4 animals) or Rho-Dend2-1D4 (wild type, based on 180 structures from *n *=* *4 animals) at 21 DPF. Scale bars:  Zoom, 1 μm; other panels, 10 μm.

Based on past studies (Li et al., 1996; Concepcion and Chen, 2010; Lodowski et al., 2013), class I mutant rhodopsin molecules are secreted as vesicles that accumulate in the interphotoreceptor space. We reproduced these findings in a *X. laevis* model that expresses class I mutant (Q344ter) rhodopsin fused to Dendra2 in rod photoreceptor cells. Rhodopsin-laden vesicles were released into the extracellular space of retina explant culture (Fig. 1*A*, arrowheads). At 9 DPF, the majority (∼93%) of rods expressing Rho_Q344ter_-Dend2 were associated with vesicles (3.45 ± 0.81 vesicles associated with a single rod, *n *=* *160 rod cells from four animals) in the extracellular space. As apical extensions of RPE cells are involved in phagocytic activities in the interphotoreceptor space, we asked whether RPE cells are capable of engulfing and clearing these vesicles. Apical microvilli and basal plasma membrane of RPE cells are densely coordinated with F-actins (Bonilha et al., 1999), which were labeled with fluorescently conjugated phalloidin (Fig. 1*B*,*C*, actin). Cytoplasmic portions of the RPE cells were barely labeled and readily recognizable from apical and basolateral borders (Fig. 1*B*,*C*, merge, double-headed arrows). In retinas expressing Rho_Q344ter_-Dend2, microvesicles were associated with the RPE microvilli (Fig. 1*B*, zoom, arrowheads), indicating these vesicles were in the process of being engulfed.

We obtained two lines of evidence that a significant fraction of microvesicles released by Rho_Q344ter_-Dend2-positive rods are engulfed by RPE cells. First, ∼40% of all vesicular structures in the cytoplasm of the RPE (Fig. 1*B*,*D*, Q344ter) had diameters within the 95% confidence interval (0.34–0.38 μm) of the size distribution demonstrated by extracellular microvesicles (Fig. 1*A*). Second, because Rho_Q344ter_-Dend2 is synthesized in rods under the regulation of the *Xenopus* rhodopsin promoter, these Rho_Q344ter_-Dend2-containing microvesicles within the RPE must have originated from rod cells. Thus, under pathologic conditions, RPE cells are capable of engulfing rhodopsin-laden microvesicles that were secreted into the interphotoreceptor space.

Under normal conditions, wild-type rhodopsin is known to be shed in OS membrane fragments, which are phagocytosed by RPE cells. Consistently, wild-type rhodopsin fused to Dendra2 (Rho-Dend2-1D4) was observed in phagosomes within RPE cells (Fig. 1*C*, asterisks). Structures containing Rho-Dend2-1D4 were significantly larger (2.89 ± 1.31 μm, *n *=* *180 structures) than the extracellular vesicles released by rods expressing Rho_Q344ter_-Dend2 in retina explant culture (Fig. 1*A*, arrowheads; 0.36 ± 0.14 μm, *n* = 180 vesicles, *p *<* *0.001). While the majority of Rho_Q344ter_-Dend2 mislocalizes to the IS PM of rod cells, a small fraction still reaches the OS, likely due to cotrafficking with endogenous rhodopsin (Concepcion and Chen, 2010). Thus, larger OS fragments exhibiting Dendra2 fluorescence were also observed in the RPE cytoplasm of animals expressing Rho_Q344ter_-Dend2 (Fig. 1*B*, merge, asterisks). We also observed large cellular fragments that are likely derived from rod photoreceptor inner segments or cell bodies (Fig. 1*B*, merge, double asterisks). Some of those fragments contained DNA and were surrounded by Rho_Q344ter_-Dend2-positive membranes, suggesting that they were rod photoreceptor cell bodies. Unlike the RPE cells of animals expressing Rho_Q344ter_-Dend2, in which 40% of vesicular structures are at the size of microvesicles, nearly all the structures in the RPE layer of animals expressing Rho-Dend2-1D4 animals were larger than microvesicles (Fig. 1*D*, compare Q344ter to wild type), confirming that wild-type rhodopsin is shed and phagocytosed as large OS fragments but not as microvesicles.

### RPE cells do not contribute to the degradation of NKA under normal physiological conditions

**Figure 2.**
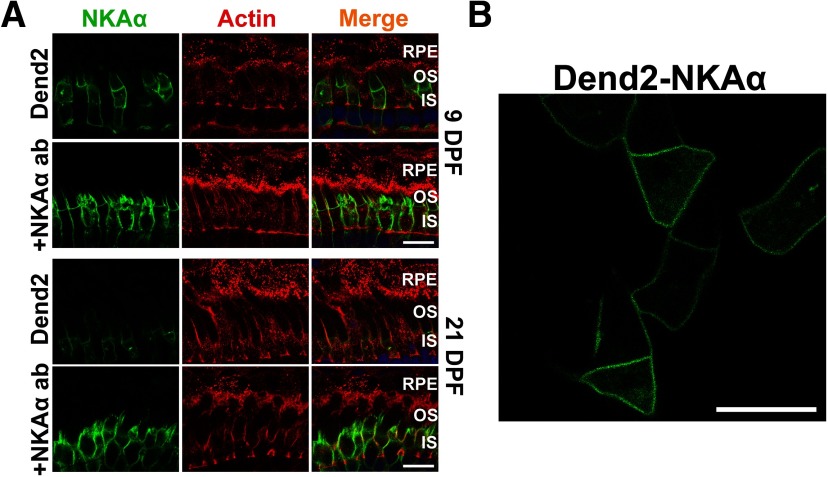
NKAα is not released in extracellular vesicles under normal physiological conditions. ***A***, Cross sections of retinas expressing Dend2-NKAα were labeled with phalloidin-Alexa Fluor 633 (Actin, red) and Hoechst 33342 dye (Nuclei, blue). Retinas were 9 DPF (top two rows) or 21 DPF (bottom two rows). For each panel, either Dend2-NKAα fluorescence was imaged directly (Dend2, green) or NKAα (both endogenous and Dend2-NKAα) was visualized by immunofluorescence (+NKAα ab, green). ***B***, Live retina explant was imaged at 9 DPF. NKAα-containing extracellular vesicles were not observed in the vicinity of rod cells expressing Dend2-NKAα. Scale bars, 10 μm.

**Figure 3.**
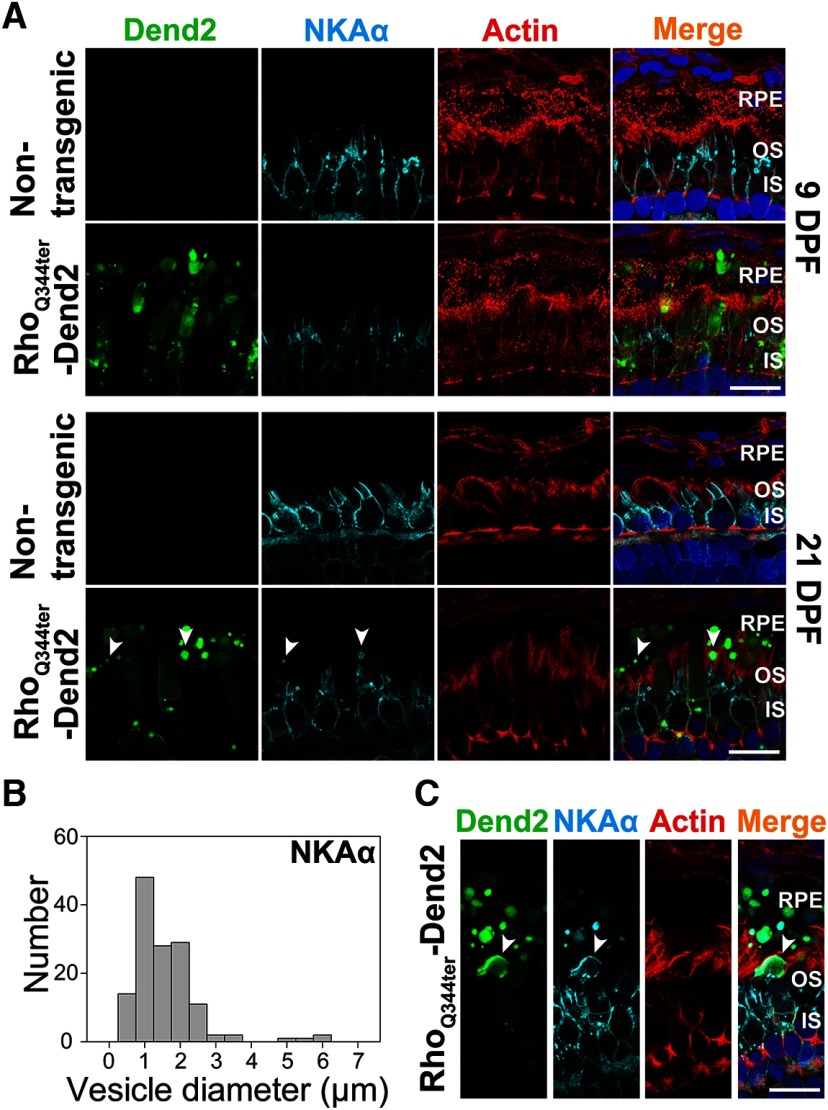
IS fragments containing NKAα are phagocytosed by RPE at a late stage of photoreceptor degeneration. ***A***, Cross sections of retinas that express either no transgenic protein (Nontransgenic) or class I mutant rhodopsin fused to Dendra2 (Rho_Q344ter_-Dend2). Retinas were 9 DPF (top two rows) or 21 DPF (bottom two rows). Sections were labeled with anti-NKAα antibody (NKAα, cyan), phalloidin-Alexa Fluor 633 (Actin, red), and Hoechst 33342 dye (Nuclei, blue). Photoreceptor-derived NKAα was not shed in microvesicles at either 9 or 21 DPF, whereas large vesicular structures (arrowheads) containing NKAα were occasionally seen in contact with RPE microvilli or inside RPE layer at 21 DPF. ***B***, Histogram indicating the size distribution of structures containing NKAα within the RPE layer. Retinas expressing Rho_Q344ter_-Dend2 were analyzed at 21 DPF. ***C***, A cross section of 21 DPF retina expressing Rho_Q344ter_-Dend2 and labeled with anti-NKAα (NKAα, cyan), phalloidin-Alexa Fluor 633 (Actin, red) and Hoechst 33342 dye (Nuclei, blue). In this view, a large inner segment fragment containing NKAα (arrowhead) is being engulfed by an RPE cell. Scale bars, 10 μm.

The above experiments suggested that IS PM mislocalized rhodopsin is packaged in microvesicles that are engulfed by RPE cells. We asked whether a similar mechanism removes IS PM resident proteins. NKA is highly expressed on the photoreceptor IS PM (Kwok et al., 2008) and is essential for maintaining the dark current (Schneider and Kraig, 1990). Thus, we focused on the degradation of the α3-subunit of NKA (NKAα), which is enriched in photoreceptor neurons (Wetzel et al., 1999). To emulate the above experiments visualizing class I mutant rhodopsin, the NKA α3-subunit was fused to Dendra2 (Dend2-NKAα) and expressed in rod photoreceptors under the regulation of *Xenopus* rhodopsin promoter. To visualize the borders of RPE cells, retinas expressing Dend2-NKAα were stained with fluorescently conjugated phalloidin (Fig. 2*A*, actin). In these retinas, Dend2-NKAα was not observed in the RPE cells (Fig. 2*A*, Dend2, RPE), indicating that the photoreceptors were not secreting Dend2-NKAα. Consistently, Dend2-NKAα was not released to the extracellular space as vesicles in live retina explant culture (Fig. 2*B*). To exclude the possibility that vesicles were present but not visible due to our limited detection sensitivities of Dendra2, we enhanced the fluorescence signal by labeling these samples with anti-NKAα (Fig. 2*A*, +NKAα ab). Regardless of rigorous labeling techniques, no vesicles were observed in the RPE layer. We obtained similar results with wild-type (nontransgenic) animals, which did not express Dend2-NKAα. In these animals, endogenous NKAα, labeled by immunofluorescence, was not observed in microvesicles extracellularly or within RPE cells (Fig. 3*A*, nontransgenic), confirming that RPE cells do not contribute to the degradation of endogenous NKAα under normal physiological conditions.

### NKAα is not cosecreted along with class I mutant rhodopsin as microvesicles

In rods expressing class I mutant rhodopsin (Rho_Q344ter_-Dend2), NKAα is downregulated because of its co-degradation with Rho_Q344ter_-Dend2 by lysosomes (Ropelewski and Imanishi, 2019). As our current study demonstrates engulfment of vesicles containing Rho_Q344ter_-Dend2 by RPE cells, we asked whether NKAα was also co-degraded by this route. In rods expressing Rho_Q344ter_-Dend2, active degradation of NKAα proteins was observed around 9 DPF (Ropelewski and Imanishi, 2019). At this early stage (9 DPF), Rho_Q344ter_-Dend2 is actively released as vesicles from rods (Fig. 1*A*) and engulfed by RPE cells (Fig. 1*B*). We used anti-NKAα antibody to label endogenous NKAα in retinas expressing Rho_Q344ter_-Dend2 at 9 and 21 DPF (Fig. 3*A*, Rho_Q344ter_-Dend2, NKAα, cyan). NKAα was not observed in Rho_Q344ter_-Dend2-positive vesicles inside the RPE cells at 9 DPF (Fig. 3*A*, Rho_Q344ter_-Dend2, RPE). Thus, NKAα is not secreted together with Rho_Q344ter_-Dend2 as microvesicles.

In later stage (21 DPF) animals expressing Rho_Q344ter_-Dend2 (Fig. 3*A*, 21 DPF, Rho_Q344ter_-Dend2), photoreceptor degeneration advances (Lodowski et al., 2013). Likely because of rod degeneration, we observed fewer vesicles (*p <* *0.001) in the retinas of older (21 DPF) animals (16.7 ± 3.3 vesicles/100 μm retina length) than in younger (9 DPF) animals (33.9 ± 5.6 vesicles/100 μm retina length). Coincidentally with the degenerative events, NKAα proteins originating from IS PM were occasionally observed in structures in contact with RPE microvilli or within the cytoplasm of the RPE (Fig. 3*A*, 21 DPF, arrowheads). These structures contained Rho_Q344ter_-Dend2, indicating they originated from rods experiencing rhodopsin mislocalization. The size of these structures (1.33 ± 0.94 μm, based on 138 structures; Fig. 3*B*) was significantly (*p <* *0.001) larger than that of the extracellular microvesicles containing Rho_Q344ter_-Dend2 (0.36 ± 0.14 μm) in retina explant culture (Fig. 1*A*). Occasionally, large NKAα-containing fragments comparable in size to a rod IS were seen in the process of being engulfed by RPE cells (Fig. 3*C*, arrowhead). Thus, the NKAα-containing structures observed at 21 DPF are likely cellular debris from ISs that degenerated as a result of Rho_Q344ter_-Dend2 expression. In retinas not expressing Rho_Q344ter_-Dend2, we did not observe photoreceptor-derived NKAα in RPE cells either at 9 or 21 DPF (Fig. 2*A*, 9 DPF and 21 DPF). In summary, RPE cells are capable of engulfing cellular debris containing IS PM protein NKAα as a result of photoreceptor degeneration, but NKAα itself is not cosecreted with class I mutant rhodopsin as microvesicles.

### NKAα is potentially degraded within rod photoreceptors

**Figure 4.**
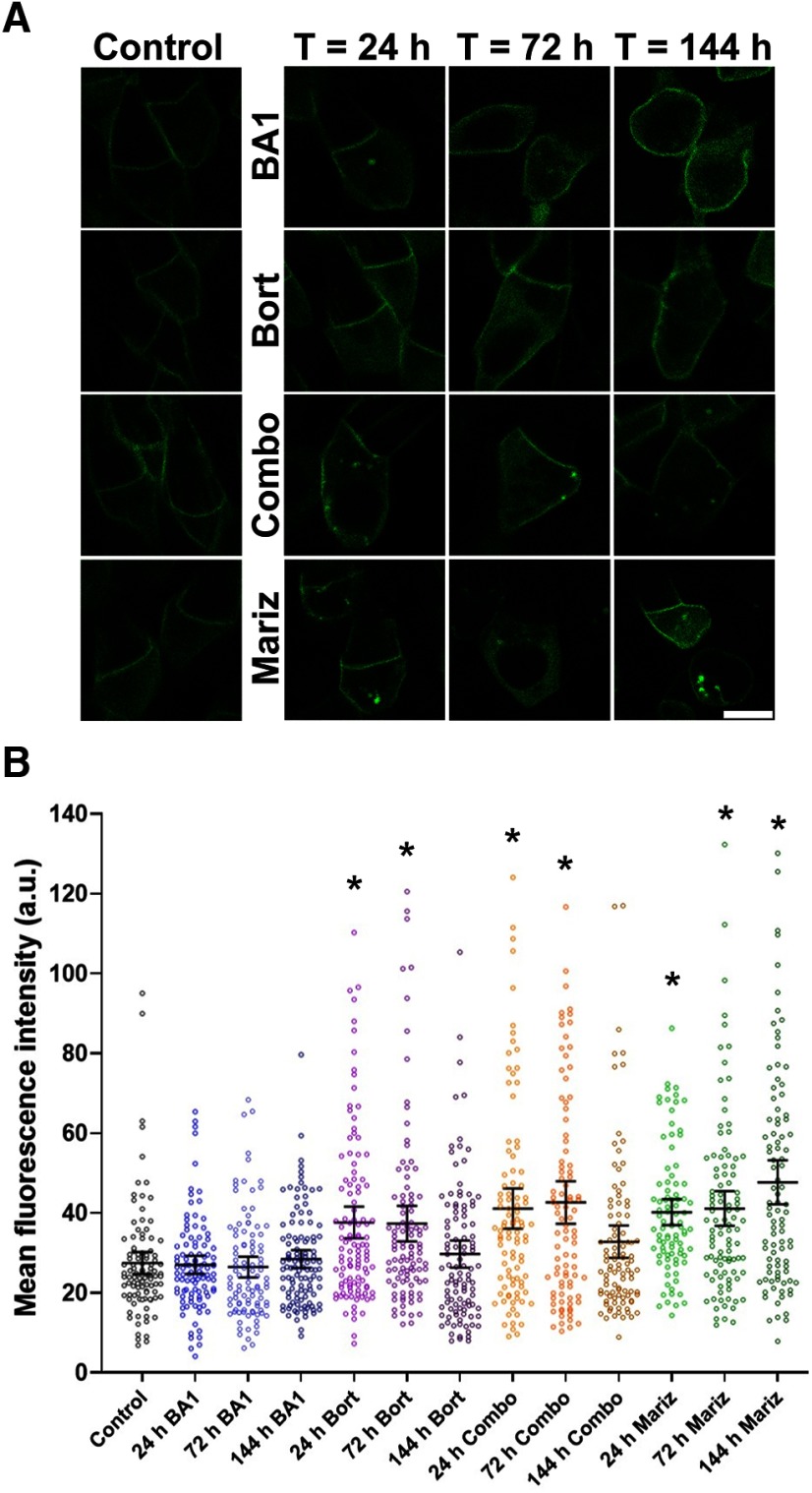
NKAα proteins are degraded intracellularly in rod photoreceptors. ***A***, Representative images of rod cells from animals expressing Dend2-NKAα and treated with either the vehicle (DMSO, control), 100 nm BA1, 100 nm Bort, a combination of both BA1 and Bort (Combo), or 250 nm Mariz for 24–144 h. Scale bar, 5 μm. ***B***, Estimation plot analysis of Dend2-NKAα concentration. Average fluorescence intensities of Dend2-NKAα were measured in the cytoplasmic region of individual rods (each dot). The error bars represent the mean ± 1.96 SEs (95% confidence interval). The average fluorescence intensities are indicated on the *y*-axis [arbitrary unit (a.u.)]. *p <* *0.001 by Student’s *t* test (each treatment condition vs control). At least 92 rod cells (*n *=* *92) from four independent animals were subjected to quantification for each condition.

IS PM protein NKAα is not removed via vesicle release and absorption by the RPE cells under normal physiological conditions. Therefore, we explored whether intracellular mechanisms contribute to the degradation of NKAα. Two potential contributors are lysosomes and proteasomes. To test the *in vivo* degradation of NKAα, we treated animals expressing Dend2-NKAα with lysosome inhibitor bafilomycin A1 (BA1) and/or proteasome inhibitor bortezomib (Bort) for various lengths of time (Fig. 4*A*). As controls, animals were treated only with the vehicle (DMSO) for 24 h (Fig. 4*A*, control). BA1 (100 nm) did not significantly increase the amount of intracellular Dend2-NKAα (Fig. 4*B*; *p *>* *0.5 for 24–144 h BA1-treated vs control), suggesting that lysosomes do not contribute to the NKAα degradation. Bort (100 nm) increased the amount of Dend2-NKAα in the IS cytoplasm significantly after 24 h (37.0% increase) and 72 h (36.1% increase) of treatment (Fig. 4*B*; *p* values < 0.001 for 24–72 h Bort-treated vs control). Similar accumulations were observed when animals received combined treatment of BA1 and Bort (Combo) for 24 h (49.6% increase) or 72 h (61.6% increase; Fig. 4*B*; *p* values < 0.001 for 24−72 h Combo vs control). We noted that the amount of intracellular Dend2-NKAα decreased after 144 h of Bort treatment (*p *<* *0.002, 72 h Bort vs 144 h Bort and 72 h Combo vs 144 h Combo). A potential reason for this decrease is resistance to Bort after long-term treatment, as characterized previously (Rückrich et al., 2009; Wu et al., 2016); this adaptive mechanism leads to an increase in proteasome subunit transcription, thus partially overcoming Bort inhibition. Therefore, we conducted an additional experiment using marizomib (Mariz), which was demonstrated to inhibit proteolytic activity even in Bort-adapted cells (Merin and Kelly, 2014). As anticipated, an increase in intracellular Dend2-NKAα was observed after 24 h (46.4% increase), 72 h (49.7% increase), and 144 h (73.7% increase) of Mariz (250 nm) treatment (Fig. 4*B*; *p* values < 0.001 for 24–144 h Mariz vs control). Considering that proteasome inhibition resulted in the accumulation of otherwise degraded Dend2-NKAα proteins, the proteasome is likely involved in the degradation of NKAα proteins. In summary, further studies are necessary to corroborate these findings because of the small effect of proteasome inhibition on protein accumulation.

## Discussion

In this article, we demonstrated that microvesicles secreted by rod photoreceptors are engulfed by and incorporated into RPE cells. These microvesicles are distinct from shed rod outer segment fragments phagocytosed by RPE cells. Under pathologic conditions such as those observed for rods expressing class I mutant rhodopsin, rhodopsin-laden microvesicles are actively shed into the interphotoreceptor space (Li et al., 1996; Concepcion and Chen, 2010; Lodowski et al., 2013). The sizes of the vesicles (95% confidence interval, 0.34–0.38 μm) in our *X. laevis* model were comparable to the sizes of vesicles (0.1–0.2 μm) in a murine model of rhodopsin class I mutation (Concepcion and Chen, 2010), indicating that the shedding of rhodopsin-laden vesicles is a conserved mechanism to expel mislocalized rhodopsin from rod photoreceptors. The past electron microscopy studies indicated that rhodopsin-laden vesicles are budding off of the IS PM in different animal models of rhodopsin mislocalization (Blanks and Spee, 1992; Hagstrom et al., 1999; Kondo et al., 2009). Previously (Lodowski et al., 2013), we used the photoconversion technique to address where microvesicles originated from in rod photoreceptor cells. In these experiments, we initially photoconverted the entire population of Rho_Q344ter_-Dend2 into red, and followed the new synthesis and trafficking of green Rho_Q344ter_-Dend2 in the cells. These experiments revealed that microvesicles, 2 d after photoconversion, were yellow in color, demonstrating a mixture of red and green isoforms of Rho_Q344ter_-Dend2. During this time frame, the IS PM had a similar composition of green and red Rho_Q344ter_-Dend2 as the microvesicles. We believe that the OS is not a source of microvesicles because such a mixture of old (red) and newly synthesized (green) protein does not occur in the OS compartment. In the OS compartment, new proteins are compartmentalized in new disks near the basal region of OSs, and old proteins are compartmentalized in the distal portion of the OSs. Six days after photoconversion, rod photoreceptors synthesized more green Rho_Q344ter_-Dend2, which was mistrafficked to the IS PM, resulting in higher green fluorescence in this compartment. Coincidentally, the microvesicles observed 6 d after photoconversion contained a higher amount of new (green) Rho_Q344ter_-Dend2. Therefore, the color composition of Rho_Q344ter-_Dend2 proteins on the IS PM is strongly correlated that of the microvesicles, indicating that the microvesicles are originating from the IS PM.

The past studies indicate that mouse models of ciliopathies caused by Tulp1 and IFT88 mutations manifest rhodopsin mislocalization and accumulation of rhodopsin-laden vesicles, as was observed in this study (Hagstrom et al., 1999; Pazour et al., 2002). Those proteins are considered essential for cilia-associated signaling and intraflagellar transport (Hagstrom et al., 2001; Nemet et al., 2015). In the Tulp1-deficient murine model, the number of rhodopsin-laden vesicles peaks at early stages (17–21 d old) when photoreceptors are still maturing (Hagstrom et al., 2001). Consistently, there were more vesicles in the early stage (9 DPF) than in the later stage (21 DPF) in our *Xenopus* model. Anatomically, the interphotoreceptor space where these vesicles are shed is accessible by RPE apical membrane extensions that are optimal for phagocytic activities. To clarify the source of the microvesicles, we fluorescently labeled the microvesicles with Rho_Q344ter_-Dend2. Because Rho_Q344ter_-Dend2 was specifically expressed in rods under the regulation of rhodopsin promoter, we concluded that fluorescent vesicles observed in RPE originated from rod photoreceptors. Engulfment of these vesicles is potentially neuroprotective as it removes rhodopsin molecules that cannot productively engage in phototransduction (Alfinito and Townes-Anderson, 2002), prevents overaccumulation of vesicular structures in the interphotoreceptor space, and potentially allows biomolecules to be recycled.

Those microvesicles did not contain NKAα, which is the major component of the photoreceptor IS PM. Thus, removal of class I mutant rhodopsin through this route will not result in the downregulation of NKAα. This is in contrast to the intracellular route of rhodopsin removal, which coincidentally downregulates NKAα (Ropelewski and Imanishi, 2019). In this route, class I mutant rhodopsin mislocalized to the IS PM is internalized together with NKAα to intracellular lysosomes, where they are codegraded, causing photoreceptor dysfunction (Ropelewski and Imanishi, 2019). To compare this behavior of class I mutant with another IS PM protein under the same condition, we expressed Dend2-NKAα in rods under the *Xenopus* rhodopsin promoter. Despite using the same expression system, we did not observe Dend2-NKAα secreted in microvesicles. Therefore, microvesicle secretion does not appear to be a normal route of degradation for IS PM-resident protein. Packaging of mislocalized rhodopsin into microvesicles is a unique cellular response to the expression of class I mutant rhodopsin, and there are specific sorting mechanisms that differentiate class I mutant rhodopsin and NKA.

To study the behavior of endogenous NKAα, we used anti-NKAα antibody, which specifically labeled NKAα in the photoreceptor IS PM layer but did not label the NKAα proteins that are localized to the apical membrane of RPE cells (Hu and Bok, 2001). Under conditions of rhodopsin mislocalization, we did not observe NKAα in the RPE layer of tadpoles at 9 DPF, when photoreceptor degeneration is not prominent and microvesicles are actively released. In later stages (21 DPF) when photoreceptor degeneration becomes apparent, we started observing structures containing NKAα around the microvilli and within the RPE cells. We also captured a large NKA-containing IS fragment in the process being engulfed by RPE cells. In line with this notion, IS components are phagocytosed by the RPE cells during photoreceptor degeneration caused by class II mutant rhodopsin (Sakami et al., 2019). Thus, these experiments confirm that endogenous NKAα proteins are not secreted as microvesicles under physiological or pathophysiological conditions. Considering that microvesicles are not removing NKAα from rods, we explored intracellular degradation pathways within rods. Using pharmacological approaches, we found that NKAα is likely degraded by proteasomes, but not by lysosomes, within rods. This is in line with our previous study in which PSmOrange-NKAα fusion protein was not degraded by lysosomes in rod photoreceptors in the absence of rhodopsin mislocalization (Ropelewski and Imanishi, 2019). Accelerated degradation of NKAα by lysosomes or proteasomes leads to its downregulation and exacerbates disease conditions such as damage of lung tissue or oxidative stress of kidney proximal tubule cells (Thévenod and Friedmann, 1999; Helenius et al., 2010). Likewise, the lysosomal pathway promotes NKAα degradation during rhodopsin mislocalization due to cotrafficking of IS PM-mislocalized rhodopsin with NKAα to lysosomes, serving as a common mechanism leading to neuronal dysfunction and degeneration (Ropelewski and Imanishi, 2019).

In summary, we demonstrated that RPE cells are capable of engulfing microvesicles released from rod photoreceptor cells. We believe that these vesicles are distinct from cilia-derived ectosomes or microvesicles described in peripherin/rds and PRCD (progressive rod-cone degeneration protein)-deficient mice (Salinas et al., 2017; Spencer et al., 2019). In these mouse models, there is no evidence of compromised rhodopsin entry into the cilia. Instead, continued entry of rhodopsin and other components is partly responsible for the bulging of the ciliary membrane and subsequent ectosome release. Moreover, RPE cells do not appear to be actively engulfing these ectosomes or microvesicles (Spencer et al., 2019). Class I mutant rhodopsin demonstrates compromised ciliary entry, similar to mouse models deficient in ciliary transport (Pazour et al., 2002). In these models, the IS PM, the site of rhodopsin accumulation, is the likely source of vesicles. This vesicle release is potentially a method to remove unwanted proteins during photoreceptor degeneration, when OS phagocytosis is compromised due to reduced OS transport of rhodopsin. Recent studies indicate that microvesicles are means of intercellular communication (Lee and Kim, 2017; Vidal-Gil et al., 2019). Contents of microvesicles, such as miRNA and signaling proteins, mediate phenotypic changes of the recipient cells (Schorey and Harding, 2016; Roballo et al., 2019). RPE cells undergo significant morphologic (Jones et al., 2016) and metabolic changes (Wang et al., 2016) in diseased retinas. In this study, we tracked microvesicles by fluorescently labeling them. This improved technique would be useful for identifying and purifying those photoreceptor-derived vesicles, which then would allow analysis of their components and further our understanding of intercellular communication in the pathologic retina.

## Synthesis

Reviewing Editor: Kirill Martemyanov, The Scripps Research Institute Decisions are customarily a result of the Reviewing Editor and the peer reviewers coming together and discussing their recommendations until a consensus is reached. When revisions are invited, a fact-based synthesis statement explaining their decision and outlining what is needed to prepare a revision will be listed below. The following reviewer(s) agreed to reveal their identity: Theodore Wensel.

Upon discussion the reviewers agreed on the significance of the work and the technical quality for the most part and as such support the publication. Reviewers also thought that revisions cold significantly improve the manuscript. Most importantly, there was a consensus that an effort to quantify phenotypes observed would particularly strengthen the conclusions. In particular, beefing up the degradation part of the story hashing out lysosomal vs proteasomal contributions if not by further experimentation then at least by consistent and more quantitative analysis and thorough discussion of limitations would be required. Overall, we feel that the manuscript could be much improved by more thorough and quantitative analysis of the existing data. Please refer to specific reviewers’ comments below.

Reviewer #1

This manuscript describes results of a study of differential processing of mutant rhodopsin and a resident protein of the inner segment plasma membrane, Na/K ATPase in the presence and absence of the mutant rhodopsin, using from transgenic frogs expressing fluorescent protein fusions and fluorescence staining of endogenous proteins.

The results show convincingly that the rods expressing the mutant rhodopsin expel microvesicles containing rhodopsin but not containing the ATPase. They also show that the microvesicles are taken up and presumably degraded by the adjacent retinal pigment epithelial (RPE) cells, although the degradation was not tested in the work presented. They further show that the ATPase is subject to proteolytic turnover primarily mediated by the proteasome and not lysosomes.

The results are interesting because they provide new insights into rod cell proteostasis and into how aberrant forms of rhodopsin, which are linked to the human blinding disease retinitis pigmentosa, are handled by rod cells and by the RPE.

The experiments are conceptually simple, the data are of good quality and the interpretation is sound. The one suggestion would be to add to some of the figures with really tiny vesicles marked by really tiny arrows as insets on an expanded scale.

Reviewer #2

Several published studies have documented the presence of extracellular vesicles in animal models of photoreceptor degeneration. In this study, authors show that RPE can engulf these vesicles. This is an interesting finding and suggests a potential pathway to reduce toxic levels of opsin. However, I have several concerns regarding the proposed mechanism for the generation of microvesicles elaborated below:

In the significance statement, authors claim that “vesicular secretion of IS PM proteins appears to be activated only under pathological conditions”. This is a strong statement as NKA appear in RPE after 21 DPF when as stated by authors RPE cells engulf cellular debris. There is no evidence of vesicular secretion of IS PM proteins.

“Vesicles were frequently observed”...Frequent doesn’t provide a quantitative measure.

Could authors comment on the difference between “microvesicles” and previously observed “ciliary ectosomes” in peripherin mutants?

What are the structures that is stained in RPE (area marked with double arrowheads) with Hoechst surrounded by RhoQ344ter staining in Fig. 1B (9DPF)? Are these glial cells?

Figure 1 title: Are all opsin mutants released as microvesicles engulfed by RPE?

Related to this question, how are authors sure that “a unique sorting mechanism” segregates opsin into the microvesicles. What could be the mechanism behind this?

The proof for IS-PM opsin packaged as microvesicles and engulfed by RPE is weak. It is also possible that opsin laden microvesicles originate from OS not from IS where NKA resides. However, the authors seem to attribute this phenomenon for the presence of larger vesicles found in the RPE.

How were the diameters of vesicles with irregular shapes calculated?

How much of opsin is degraded intracellularly in the IS versus the microvesicles?

At 144h, there is a reduction in cytoplasmic NKA levels. Is there a reason for this?

The data supporting NKA degradation by proteasome is weak and uses a single inhibitor at 100nM concentration. Quantitative measures of NKA using image analyses should be supported by western blotting.
